# Application progress of liquid biopsy in gastric cancer

**DOI:** 10.3389/fonc.2022.969866

**Published:** 2022-09-15

**Authors:** Xiaoting Ma, Kai Ou, Xiu Liu, Lin Yang

**Affiliations:** Department of Medical Oncology, National Cancer Center/National Clinical Research Center for Cancer/Cancer Hospital, Chinese Academy of Medical Sciences and Peking Union Medical College, Beijing, China

**Keywords:** ctc, ctDNA, ctRNA, exosome, gastric cancer, liquid biopsy

## Abstract

Gastric cancer (GC) is one of the most common malignant tumors globally. Guiding the individualized treatment of GC is the focus of research. Obtaining representative biological samples to study the biological characteristics of GC is the focus of diagnosis and treatment of GC. Liquid biopsy technology can use high-throughput sequencing technology to detect biological genetic information in blood. Compared with traditional tissue biopsy, liquid biopsy can determine the dynamic changes of tumor. As a noninvasive auxiliary diagnostic method, liquid biopsy can provide diagnostic and prognostic information concerning the progression of the disease. Liquid biopsy includes circulating tumor cells, circulating tumor DNA, circulating tumor RNA, tumor educated platelets, exosomes, and cytokines. This article describes the classification of liquid biopsy and its application value in the occurrence, development, and therapeutic efficacy of GC.

## Introduction

Gastric cancer (GC) is one of the most common malignant tumors. It ranks fifth in incidence globally (1) and third concerning the cancer mortality rate (1). GC is a serious threat to health and life. In recent years, the individual diagnosis and treatment of tumors has achieved good results and improved the prognosis of patients. However, due to the lack of specific markers and effective treatment methods, the recurrence and metastasis rate of GC patients is still high. Traditional methods, such as serum tumor markers, imaging endoscopy, and histopathology, are still widely used in the screening of GC. However, their sensitivity and specificity are relatively low. Liquid biopsy is an *in vitro* diagnostic technology that uses circulating biomarkers in the body fluid of tumor patients to provide tumor genetic information. It represents a rapid, non-invasive, and reproducible alternative to tissue biopsy (2, 3). With the advancement of precision treatment, liquid biopsy has greatly promoted the development of precision medicine in clinical practice on the basis of scientific research. This article reviews the application progress of liquid biopsy in GC.

## Liquid biopsy

Liquid biopsy is defined as the sampling and analysis of non-solid biological tissues, such as blood, and other body fluids, including urine, saliva, pleural fluid, and cerebrospinal fluid. The principle of the technique is that tumor will release DNA, RNA and other fragments into the blood circulation system or body fluid during cell apoptosis or necrosis. These fragments often contain the molecular information of the tumor, and their abundance is closely related to the size and development of the tumor.

**Figure 1:**
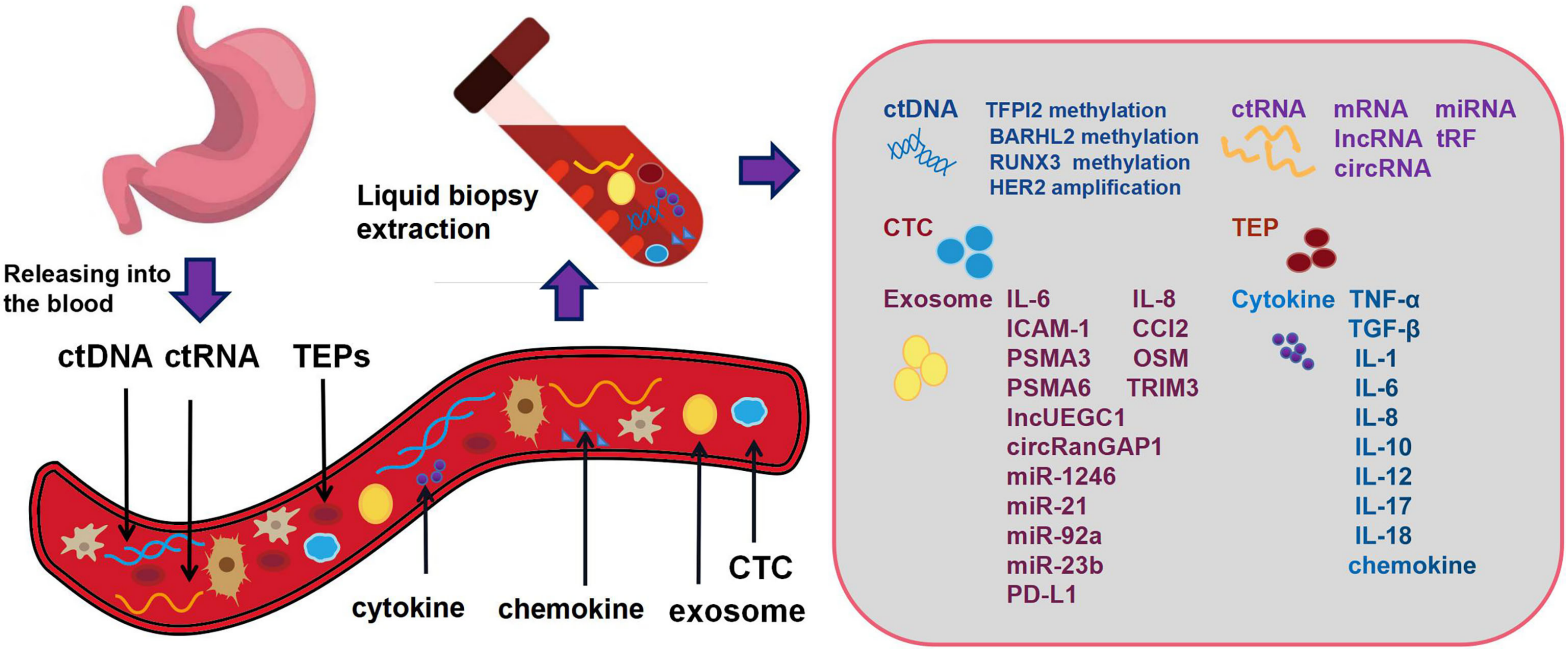
Gastric cancer cells release DNA, RNA and other fragments into the blood circulation system or body fluid during cell apoptosis or necrosis. These fragments often contain the molecular information of the tumor. Blood based liquid biopsy includes proteins and cytokines detected in plasma, circulating tumor cells, circulating tumor DNA, circulating tumor RNA, tumor induced platelets and exosomes.

Compared with the traditional tumor tissue biopsy technology, liquid biopsy has additional advantages of fast sampling speed, low cost, minimal invasion, and the ability to track tumor progression. More importantly, tumor tissue biopsy cannot be performed without knowing the tumor in advance, while liquid biopsy can identify unknown lesions. It can also monitor tumor treatment by detecting minimal residual disease and can even be used for cancer screening in healthy people. Blood based liquid biopsy includes proteins and cytokines detected in plasma, CTCs/circulating tumor microemboli (CTM), exosomes, tumor induced platelets (TEPs), and tumor derived circulating nucleic acids, such as circulating tumor DNA (ctDNA) and circulating tumor RNA (ctRNA) (**Figure 1**).

## CTCs/CTMs and GC

### CTCs and CTMs

In 1869, Ashworth discovered the existence of CTCs in the blood circulation. After being shed from the primary tumor or metastatic lesion, CTCs are released into the peripheral blood through blood vessels and lymphatics. During the development and progression of tumors, most tumor cells entering peripheral blood are cleared by immune system or die due to the shear force of blood flow. Only a small portion of tumor cells with stem cell-like or epithelial mesenchymal transition (EMT) characteristics can survive and migrate to distant sites (4, 5). Because there are very few CTCs in blood, it is very difficult to detect and count CTCs produced by malignant tumors. There are many existing technologies for detecting CTC. These mainly include enrichment and identification technology (6). The principle of enrichment technology is mainly based on the different physical or biological characteristics of CTCs from other cells in blood. CTCs are screened out by specific proteins expressed on the cell surface or by cell size and density. The enrichment methods mainly include density gradient centrifugation, immunomagnetic bead sorting, and chip enrichment technology. Among them, immunomagnetic bead sorting method is used in most detections at present because of its high sorting specificity and good activity in enriching CTCs (7). CTCs enriched by immunoaffinity or physical property need to be combined with effective analysis methods. Since the current CTC capture technology cannot guarantee 100% purity, the obtained cells need to be identified to further determine the number of CTC cells, so as to reduce the false positive rate and false negative rate of CTC number determination. In addition, in the process of tumor development, not only the number of CTCs is changing dynamically, but also the molecular markers carried by CTCs are changing. Therefore, the detection of CTC surface markers can reflect the dynamic changes of tumor occurrence and development, and can better guide clinical treatment. At present, the commonly used CTC identification technologies include immunofluorescence, PCR, FISH and high-throughput sequencing. And epithelial cell adhesion molecules and cytokeratin (CK) are the most commonly markers in the identification of CTCs (8). At present, there are also single-cell analysis technologies for CTC. First, single cells are captured and recovered by manual aspiration under the microscope and micro cutting on the slide, and then whole genome amplification is carried out. However, due to the high technical threshold of CTC single-cell analysis, it is generally necessary to combine high-performance microscopy to confirm cells (9). CTCs have proven prognostic value in different tumors, including digestive system tumors. In addition, information concerning gene mutation and protein expression in CTCs is also another important marker of tumor screening, treatment response assessment, and survival prediction.

A CTM is a cell mass formed by the aggregation of at least three cells that circulates in the blood. CTM reflects the collective migration behavior of tumor cells. Compared with CTC, CTM may be more likely to survive the shear pressure of blood flow. In addition, CTM can resist anoikis and immune clearance, overcome cytotoxic treatment, and maintain proliferation ability, which bestows a greater survival advantage and metastasis potential on the aggregate compared to a single tumor cell (10–13). CTM avoid anoikis by loss of adhesion of the tumor cells. The cells more easily metastasize through EMT transformation and maintain the internal cell connection to resist anoikis and apoptosis (14). CTM can be composed of tumor cells and can also adhere to a mixture of leukocytes, endothelial cells, parietal cells, and platelets in continuous circular collision to form a closed microenvironment. A CTM containing fibroblasts and endothelial cells is more likely to avoid immune clearance and form distal metastatic lesions (10). RNA sequencing analysis also suggests that CTM contains key molecules to maintain cell adhesion, and that the CTM-related microenvironment is conducive to tumor survival. Once CTM forms, tolerance and proliferation rate are greatly improved. In one study, a frequency of CTMs ≥ 1 was associated with a higher risk of disease progression and death, as well as shorter progression-free survival (PFS) and overall survival (OS) (15). Therefore, although the number of CTMs is less, their metastatic tumorigenicity is 23−50 times higher than that of a single CTC. Therefore, the formation of CTMs is generally considered to lead to a worse prognosis.

### CTCs/CTMs and occurrence, development, and therapeutic efficacy of GC

The positive rate of CTCs in peripheral blood of patients with GC is significantly higher than that of patients with benign gastric diseases (16). A meta-analysis showed that the specificity of CTC detection for GC diagnosis was 99% (17). Cao et al. detected CTCs in the peripheral blood of GC patients following surgery by detecting the expression of *Survivin*. The authors described that the detection rate of CTCs in advanced GC (AGC; stage III ~ IV) was significantly higher than that in stage I~II (18). However, the definition of a CTC-positive standard has not been unified. Kang et al. conducted a prospective study including 116 patients with GC and 31 healthy subjects. The receiver operating characteristics (ROC) curve showed that when the CTC threshold was 2 per 7.5 mL of blood, the sensitivity and specificity of CTC in distinguishing GC from normal controls were highest (85.3% and 90.3%, respectively) (19). These results implicate CTCs as a potential biomarker for early diagnosis of GC.

Compared with conventional tumor markers, monitoring CTCs is more sensitive for the assessment of disease status and prognosis of GC. Cheng et al. found that the CTC count of AGC patients significantly correlated with many clinicopathological parameters, including Lauren grade, peripheral nerve infiltration, TNM stage, and Ki-67 level (20). Several other studies have confirmed the significant correlation of CTCs with the survival of patients. Hiraiwa et al. used the CellSearch system to detect the CTC level of 44 GC patients; the OS of patients with CTC < 2 was significantly better than that of patients with CTC ≥ 2 (21). The positive rate of CTC in metastatic GC (mGC) was as high as 61%, which was significantly higher than that in patients with non-mGC. Uenosono et al. detected CTCs of 251 GC patients using the CellSearch system. The recurrence free survival (RFS) and 5-year survival rates of CTC-positive patients were significantly lower than those of CTC-negative patients (22). In addition, in a study involving 41 patients with newly diagnosed mGC, Zheng et al. tested the prognostic significance of CTM in peripheral blood samples. In multivariate analysis, detectable CTM in blood was an independent prognostic factor for shortened OS (23). The collective evidence supports the view that CTCs/CTMs could be used as indicators to judge the prognosis and recurrence of GC.

CTC is also important in monitoring the efficacy of GC. Lee et al. found that among patients with mGC, patients with CTC-positive (≥ 5 CTCs per 7.5 mL) had lower chemotherapy efficacy and reactivity than patients with CTC-negative (< 5 CTCs per 7.5 mL) (24). Li et al. monitored the dynamic changes of CTCs during treatment of 15 AGC patients. Patients with sustained low level CTC (< 3 CTCs per 7.5 mL) or patients who changed to low-level CTC early after treatment had a better prognosis. In contrast, patients with sustained high-level CTC (≥ 3 CTCs per 7.5 mL) or patients who changed to high level CTC after treatment had a worse prognosis (25). Matsusaka et al. found that the number of CTCs in peripheral blood of AGC patients decreased significantly after 2 and 4 weeks of treatment with tegafur, gimeracil, and oteracil potassium capsules, especially after 2 weeks of chemotherapy (26). Therefore, regular detection of CTCs may guide the clinical use of chemotherapeutic drugs. The reduction of CTCs is an early indicator of effective treatment.

## The ctDNA and GC

### The ctDNA

Circulating free DNA (cfDNA) is a kind of extracellular DNA that can be present in blood, cerebrospinal fluid, and other body fluids in the form of single stranded or double stranded DNA, and as a complex of DNA and protein. The cfDNA fragments are released by cells into the blood. The term cfDNA refers to DNA released by tumor cells into the blood. Because ctDNA contains different fragments of tumor genes, which may contain cancer specific genes or epigenetic variations, such as methylation or mutation. The average length of ctDNA is approximately 134bp, which is shorter than that of non-tumor cfDNA (approximately 166bp) (27). The difference in length can separate ctDNA from non-tumor cfDNA. Compared with CTC, ctDNA has a relatively long half-life, which confers an advantage in reflecting tumor heterogeneity.

### The ctDNA and GC occurrence, development, and therapeutic efficacy

The ctDNA can be used as a tumor marker for early diagnosis of GC. Kim et al. confirmed that the ctDNA level of GC patients was higher than that of healthy controls. The diagnostic sensitivity and specificity was 96.67% and 94.11% respectively (28). Park et al. studied 54 GC patients and 59 healthy controls, and found that the concentration of ctDNA in the GC patients was 2.4 times higher than that in the healthy controls (29). Sai et al. detected the ctDNA concentration of 53 GC patients by real-time fluorescence quantitative PCR (RTQ-PCR). The ctDNA concentration of GC patients was significantly higher than that of healthy controls, and that of patients with AGC was higher than that of patients with early GC (EGC), indicating that the ctDNA concentration was related to the occurrence and development of GC (30). The methylation level of BARHL2 appears to be helpful in distinguishing GC and non-GC individuals. Another study detected TFPI2 methylation of tumor cells in the plasma of 73 GC patients. Real-time fluorescence quantitative methylation specific PCR detected TFPI2 methylation in 7 patients, but not detected in the healthy controls. Analysis of the samples by RTQ-MSP revealed that the degree of TFPI2 methylation was higher in patients with lymph node and distant metastases. These findings suggest that TFPI2 methylation is related to the occurrence and metastasis of GC (31). A meta-analysis of 1193 GC patients showed that the detection of ctDNA had significant advantages in the specificity of diagnosis of GC, and was significantly correlated with tumor size, TNM stage and infection with *Helicobacter pylori* (32).

The effect of ctDNA on the prognosis and treatment of GC patients has been gradually confirmed. Gao et al. showed that the detection of ctDNA was significantly correlated with the TNM stage of GC. PFS and OS of patients with detectable ctDNA were significantly shorter than those without detectable ctDNA (32). Another study associated ctDNA levels with tumor recurrence in GC patients undergoing therapeutic surgery (28). Kim et al. monitored the ctDNA in GC patients postoperatively and detected ctDNA was detected in 19 samples. The average time from the detection of ctDNA to the recurrence of GC was 4.05 months. These authors reported that the presence of postoperative ctDNA was significantly correlated with the recurrence of GC within 12 months after operation, indicating the clinical significance of postoperative ctDNA monitoring for the recurrence of GC in patients (33). Yang et al. observed 46 patients with stage I-III GC postoperatively; relapse eventually occurred in all patients with detectable ctDNA immediately after surgery (34). Sakakura et al. used RTQ-MSP to detect RUNX3 methylation of ctDNA in GC patients. RUNX3 hypermethylation was detected in 29% (19/65) GC patients. The postoperative level of RUNX3 methylation was 12-times lower than the preoperative level. A prospective study of the RUNX3 methylation level in all preoperative and postoperative GC patients found that the postoperative level decreased by 83% compared with the preoperative level, and that the degree of RUNX3 methylation was related to tumor stage, lymph node metastasis, and vascular invasion, with a sensitivity higher than that of carcinoembryonic antigen (CEA) (35). In addition, the research results of Wu et al. concerning the chromosomal instability and treatment response of ctDNA in GC showed that 93% of patients sensitive to drug treatment had stable chromosomes, while 52% of patients resistant to treatment had no stable chromosomes. These findings indicate that the detection of chromosomal instability of ctDNA may be helpful to monitor the treatment response of GC patients (36).

Recent studies have also highlighted more advantages of ctDNA. Maron et al. showed that, compared with tissue-next generation sequencing (NGS), ctDNA-NGS can overcome heterogeneity and identify a higher frequency of gene mutations (37). Gao et al. analyzed the amplification of HER2 in ctDNA-NGS of 70 GC patients. The total coincidence rate between the results and immunohistochemistry/fluorescence *in situ* hybridization in tissues was 91.43%. This finding indicated a high consistency between ctDNA and tissue biopsy for the detection of HER2 amplification. Gao et al. further compared the detection of ctDNA and tumor tissue in 30 patients with AGC. They considered that the evaluation of ctDNA could partially overcome the heterogeneity of tumor and might become a substitute for HER2 analysis in GC (38). The collective findings indicate that the detection of ctDNA can be used for the early diagnosis, treatment, and prognostic evaluation of GC (**Table 1**).

**Table 1:** Biological functions of liquid biopsy in GC.

Liquid biopsy	Tendency	Downstream signal/target	Function
BARHL2 methylation	up	NA	Tumor population differentiation
TFPI2 methylation	up	NA	Promote invasion and metastasis of GC
RUNX3 methylation	up	NA	Promote metastasis and vascular invasion of GC
HER2 amplification	up	NA	Replacement of tissue biopsy
miR-10b-5p	up	NA	Early diagnosis of GC
miR-20a-3p	up	NA	Early diagnosis of GC
miR-132-3p	up	NA	Early diagnosis of GC
miR-185-5p	up	NA	Early diagnosis of GC
miR-195-5p	up	NA	Early diagnosis of GC
miR-296-5p	up	NA	Early diagnosis of GC
miR-16	up	NA	Early diagnosis of GC
miR-25	up	NA	Early diagnosis of GC
miR-92a	up	NA	Early diagnosis of GC
miR-451	up	NA	Early diagnosis of GC
miR-486-5p	up	NA	Early diagnosis of GC
miR-212	down	NA	Inhibit invasion and metastasis of GC
miR-196a	up	NA	Early diagnosis of GC
miR-30a-5p	up	NA	Poor prognosis
miR-659-3p	up	NA	Poor prognosis
miR-3917	up	NA	Poor prognosis
miR-203	up	NA	Promote invasion and metastasis of GC
lncRNA H19	up	NA	Promote GC cell growth and inhibit apoptosis
lncRNA MALAT1	up	NA	Independent risk factors for OS
circNRIP1	up	AKT1/mTOR	Promote invasion and metastasis of GC
tRF-3017A	up	Silencing NELL2	Promote invasion and metastasis of GC
tRF-5026a	down	PTEN/PI3K/AKT	Inhibit occurrence and development of GC
tRF-Glu-TTC-027	down	MAPK signaling	Inhibit occurrence and development of GC
Exosomal lncUEGC1	up	NA	Early diagnosis of GC
Exosomal circRanGAP1	up	VEGFA	Promote invasion of GC
Exosomal miR-1246	up	NA	Early diagnosis of GC
Exosomal miR-21	up	NA	Early diagnosis of GC
Exosomal miR-92a	down	NA	Better OS和PRFS
Exosomal miR-23b	up	NA	Independent risk factors for DFS and OS
Exosomal PD-L1	up	CD69	T cell dysfunction

NA, not applicable.

## The ctRNA and GC

### The ctRNA

The ctRNA is present in the blood circulation and has some potential as a cancer biomarker. Compared with ctDNA, ctRNA is unstable and has a short half-life, and its wide classification and distribution in peripheral blood and other body fluids make it a superior biomarker for liquid biopsy. At present, mRNA fragments and microRNAs (miRNAs) are usually the main detection targets of ctRNA. In addition, long noncoding RNA (lncRNA), circular RNA (circRNA), and transfer RNA (tRNA) derived fragment (tRF) are also new biomarkers in cancer diagnosis and treatment monitoring (39–41).

### The ctRNA and GC occurrence, development, and therapeutic effect

Studies of several tumor species, including GC, have confirmed the role of ctRNA in tumor diagnosis and prognosis. Due to their abundance, stability, tissue-specific expression and wide circulation in different body fluids and exosomes, ctRNA may become a new diagnostic biomarker. MiRNAs are currently considered as promising GC biomarkers. These RNAs are abnormally expressed in precancerous events of gastric tissue, such as *H. pylori* infection and precancerous lesions, including chronic atrophic gastritis and intestinal metaplasia, and in early and advanced GC (42). The expression of miR-21 and mIR-19 increased, and the expression of let-7, miR-146, and miR-375 decreased in GC patients infected with *H. pylori*, indicating the potential of miRNA as a biomarker for early diagnosis of GC (43). Up-regulation of miR-10b-5p, miR-20a-3p, miR-132-3p, miR-185-5p, miR-195-5p, and miR-296-5p in the serum of patients with GC has been demonstrated (44). Up-regulation of miR-16, miR-25, miR-92a, miR-451, and miR-486-5p in the plasma of patients with GC can diagnose GC in the early stage (45). Shao et al. demonstrated the significantly lower expression of miR-212 in GC patients than that in healthy controls, and was negatively correlated with tumor stage (46). Another study showed that miR-196a had higher diagnostic ability than miR-196b or miR-196a/b combination, which also confirmed its potential value as a biomarker of GC (47). In addition, Shimura et al. reported the overexpression of miR-30a-5p, miR-659-3p, and miR-3917 in GC patients with peritoneal metastasis, and demonstrated that the high expression of these miRNAs was significantly correlated with poor prognosis (48). Imaoka et al. showed that the low level of serum miR-203 in GC patients was related to lymph nodes, peritoneum, and distant metastasis, which eventually led to a poor prognosis (49).

The expression of circulating lncRNAs is also related to the diagnosis, prognosis, and treatment monitoring of GC patients. The authors described that plasma lncRNA H19 in GC patients was higher than that in non-GC patients (50, 51). LncRNA H19 can promote the growth of GC cells and inhibit apoptosis (52). Dai et al. found that lncRNA MALAT1 was highly expressed in GC; high expression of MALAT1 was an independent risk factor for OS in GC patients (53). In addition, MALAT1 can promote the malignant progression of GC and resistance of GC cells to cisplatin, implicating MALAT1 as a potential biomarker for predicting the prognosis of GC. Advantages of circRNA include richness, stability, and tissue specificity, and they is widely circulated in a variety of body fluids and exosomes. Therefore, circRNA may become a new GC diagnostic biomarker and therapeutic target (54). RNA sequencing analysis has shown that circRNA was highly specifically expressed in GC, and its expression level was closely related to the malignant biological behavior of GC. Zhang et al. confirmed by qRT-PCR that the expression of circNRIP1 in GC cells was significantly higher than that in normal gastric mucosal epithelial cells. The expression of circNRIP1 can activate protein kinase B (AKT)1/mammalian target of rapamycin signaling pathway by binding miR-149-5p to promote the proliferation, invasion, and migration of GC cells (55). In addition, different derived fragments of tRNA have different roles in the occurrence and development of GC. Tong et al. showed that tRF-3017A could promote the migration and invasion of GC cells by silencing tumor suppressor NELL2 (56). The tRF-5026A and tRF-Glu-TTC-027 can inhibit the occurrence and development of GC through the phosphatase and tensin homolog (PTEN), phosphoinositide 3-kinase (PI3K), AKT, and mitogen-activated protein kinase (MAPK) signaling pathways, respectively (57, 58) (**Table 1**).

## Exosomes and GC

### Exosomes

Exosomes are the most widely studied of the three major subgroups of extracellular vesicles (EV) released from mammalian cells (the others are microcysts and apoptotic vesicles) (59). Almost all cells in the body, including tumor cells, can produce exosomes. These EVs can carry a variety of proteins, mRNA, miRNA, lncRNA, DNA, and lipids. The surface molecules are composed of integrin and the Tetraspanins family. CD9, CD63, and CD81 are often used as specific markers of exosomes (60, 61). Exosomes are widely prevalent in urine, blood, pleural and peritoneal effusion, saliva, bile, and semen. In particular, they are secreted by tumor cells, which may be related to Rab3D overexpression, Wnt signal pathway activation, and an acidic microenvironment (62–64). Recent studies have shown that tumor derived exosomes carry a large number of functional molecules and have become a new model of intercellular signal transduction media. They initiate intercellular information communication by fusing with the target cell membrane and transmit functional molecules, including miRNA and protein. In addition, exosomes participate in a series of processes that include immune response, cell migration, cell proliferation, cell differentiation, and tumor invasion. Exosomes promote tumor growth and metastasis by immunosuppression, inducing EMT in tumor cells, promote angiogenesis, and enhance vascular permeability, which establishes the microenvironment before tumor metastasis and transmission of drug resistance.

### Exosomes and GC occurrence, development, and therapeutic effect

GC derived exosomes usually promote proliferation in an autocrine manner or by activating MAPK, extracellular signal-regulated kinase (ERK), PI3K, and AKT signaling pathways (65, 66). GC derived exosomes are further involved in the dissemination of tumors in the abdominal wall and diaphragm (67). A variety of exosomal proteins have been identified as new biomarkers for the diagnosis and prognosis of GC. In addition, omentum may play a positive role in promoting the invasion of GC cells through secreted proteins such as interleukin (IL)-6, IL-8, intercellular adhesion molecule 1 (ICAM-1), C-C motif chemokine ligand 2 (CCL2), and oncostatin M (OSM) (68). Ding et al. reported that the exosomal proteasome 20S subunit alpha 3 (PSMA3) and PSMA6 in mGC were significantly enriched in serum compared with primary GC, and could be potential biomarkers of mGC (69). The exosomal tripartite motif containing 3 (TRIM3) was found to be an anti-EMT factor (70). The authors described that, compared with the healthy controls, the level of TRIM3 in serum of GC patients decreased. Knockdown of TRIM3 promoted the growth and metastasis of GC by regulating stem cell factor and EMT regulatory factor. In addition, overexpression of TRIM3 inhibited the growth and metastasis of GC *in vitro* and *in vivo* (70). Because the vesicles of exosomes can stabilize the existence of genetic material, exosomes can help screen for specific genes to judge the prognosis of patients with GC.

The role of ctRNA in the occurrence and development of GC was described above. In GC, substantial RNA is released by exosomes. This released RNA is important in the diagnosis and prognosis of GC. Two specific exosomal lncRNAs, lncUEGC1 and lncUEGC2, were reportedly significantly up-regulated in the exosomes of EGC patients. In a study that addressed the differentiation of EGC from precancerous chronic atrophic gastritis, the area under the ROC curve value of lncUEGC1 was higher than that of CEA, suggesting that the diagnostic accuracy of lncUEGC1 was higher than CEA (71). Exosomal circ-RanGAP1 was elevated in plasma of GC patients and promoted GC invasion by up-regulating vascular endothelial growth factor A expression (72). Shi et al. confirmed that the increased expression of serum exosomal miR-1246 could distinguish patients with GC from healthy controls and patients with benign diseases, highlighting the use of this molecule as a biomarker for early diagnosis of GC (73). Soeda et al. explored the potential value of miR-21 and miR-92a as biomarkers from 129 patients with stage II and III GC. Compared with the healthy controls, the level of miR-21 in GC patients increased significantly, while the level of miR-92a decreased significantly. OS and peritoneal RFS of stage II and III patients with high miR-21 levels were worse than those with low miR-21 levels. The OS and peritoneal RFS of stage II and III patients with low miR-92a levels were significantly worse than those with high miR-92a levels (74). Kumata et al. considered that miR-23b was an independent prognostic factor of OS and disease free survival in each stage of GC (75).

In addition, studies on exosomes have increasingly focused on regulation of tumor immunity. Exosomal programmed death-ligand 1 (PD-L1) has been proven to be an independent prognostic factor of GC. The expression of PD-L1 is related to the immunosuppressive state and the decline of CD4 + and CD8 + T cell counts and granzyme B in GC patients (76, 77). In addition, exosomal PD-L1 reduces CD69 on the surface of T cells and causes T cell dysfunction by binding to PD-1 positive tumor associated macrophages (TAMs) (76). The collective findings indicate that exosomes participate in the immune regulation of GC by regulating cellular signaling pathways in the tumor microenvironment (**Table 1**).

## TEPs and GC

In normal individuals, platelets are the second most abundant cells after red blood cells (78). Platelets also play important roles in tumor growth and migration. Heeke et al. found that platelets could assist the immune escape of tumor cells and ultimately enhanced tumor escape and angiogenesis (79). In addition, platelets can release many cytokines, including transforming growth factor-beta (TGF-β). Platelets promote EMT and enhance the invasiveness of tumor cells. Radziwon et al. confirmed that platelets could enhance the activation and expression of matrix metalloproteinase-9 (MMP-9) through the p38 MAPK pathway, in turn enhancing the invasiveness of tumor cells (80). In 2015, Best et al. proposed the concept of TEPs (81). Platelets are rich in RNA. During the occurrence and development of cancer, tumor cells and tumor microenvironment interact with platelets indirectly through different signal molecules or directly through different receptors, such as P-selectin. The interaction changes the information of RNA and protein of platelets. Platelets whose RNA expression is changed due to the influence of tumors are termed TEPs. Although the biological mechanism leading to the change of RNA expression of TEPs is still unclear, the RNA expression of TEPs is dynamically affected by pathophysiological conditions in tumor patients, and TEPs can promote the invasion and metastasis of tumor cells and encapsulate CTC to escape the immune mechanism. Thus, TEPs can enhance the early diagnosis of tumors and are a good biomarker for tumor surveillance (82). Saito et al. showed that the platelets of GC patients can promote the malignant behavior of GC cells through EMT related signaling or direct contact with tumor cells (83). Other studies have shown that the activity and quantity of platelets can be increased in patients with GC. Activated platelets express CD40L and CD62P on the cytoplasmic membrane and interact with vascular endothelium to induce the production and metastasis of tumor growth factor (84, 85). This may all be related to the effect of TEPs. At present, there are few studies on TEPs in GC. However, because the unique miRNA expression profile of TEPs is conducive to the diagnosis of tumors, TEPs may well become a detection marker of tumors, including GC.

## Cytokines and GC

The chronic inflammatory response is important in the occurrence and development of GC. This involves cell transformation, survival, proliferation, invasion, angiogenesis, and metastasis. Because *H. pylori* can specifically survive in acidic environment, adhesion of these bacteria to gastric mucosa can induce an immune response, with the successive recruitment of concentrated granulocytes, B lymphocytes, T lymphocytes, and macrophages (86, 87). The release of inflammatory factors, including IL-1β, IL-1 receptor antagonist, IL-8, IL-10, and tumor necrosis factor-alpha (TNF-α) changes the adhesion of gastric mucosal cells, which leads to the migration and diffusion of tumor cells without mutations of tumor suppressor genes.

### TNF-α

TNF-α is an inflammatory mediator produced by leukocytes, tumor cells, and other cells in the tumor microenvironment. It is involved in regulating the growth and differentiation of a variety of cells, and is also related to the occurrence and development of tumors. The biological effects of TNF-α are mediated by two transmembrane TNF receptors (TNFR) on the cell surface. TNFR1 is expressed in a variety of cells, while TNFR2 is mainly expressed in epithelial cells and immune cells. The affinity of TNFR2 for TNF-α is much higher than that of TNFR1, but TNFR1 is the main functional receptor (88).

The activation of Wnt/β-catenin signaling pathway plays a very important role in the occurrence of gastrointestinal malignant tumors. Song et al. reported that TNF-α induced the expression of Wnt10a and Wnt10b in GC cells and further activated the Wnt/β-catenin/T cell factor signaling pathway, which induced GC (89). Oshima et al. found that TNF-α/TNFR1 signaling promoted the occurrence of GC by inducing the expression of Noxo1 and Gna14 in tumor cells. In addition, TNF-α can promote the migration and invasion of GC cells (90). Guo et al. found that TNF-α was an independent risk factor for peritoneal metastasis, which was positively correlated with tumor size and depth of invasion, and negatively correlated with the degree of tumor cell differentiation (91). Oku et al. showed that TNF-α up-regulated the secretion of MMP-9 by GC cells and peritoneal mesothelial cells, which promoted the peritoneal dissemination of cancer cells (92). Bigatto et al. confirmed that TNF-α induced MET transcription through nuclear factor-kappa B (NF-κB) and maintained MEK/ERK activation and snail accumulation through MET, resulting in the down-regulation of E-cadherin and cell migration (93). Claudin 1 is a tightly connected component with a variety of biological functions that include promotion of cell proliferation, migration, and invasion. MNK-28 cells exposed to TNF-α are induced to express claudin 1 expression and cell migration is promoted. Knock-out of claudin 1 significantly weakens the ability of TNF-α to enhance MNK-28 cell migration. This finding indicates that the migration ability of GC cells may be changed by claudin 1, downstream signal molecule of TNF-α (94). Since TNF-α is closely related to the formation of GC, TNF-α may be one of the markers for its diagnosis, efficacy, and survival (**Table 2**).

**Table 2:** Biological functions of cytokines in GC.

Author	Cytokine	Signal/target	Function	Tendency
Song [85]	TNF-α	Activate the Wnt/β-catenin signaling pathway	Induce the generation of GC stem cells	up
Oshima [86]	TNF-α	TNF-α/TNFR1 → Noxo1/Gna14	Maintain tumor cells in undifferentiated state	up
Oku [88]	TNF-α	Induce MMP-9 expression	Promote GC invasion and metastasis	up
Bigatto [89]	TNF-α	Activate NF-κB → EMT→ MEK/ ERK activation → E-cadherin down-regulated	Promote GC invasion	up
Shiozaki [90]	TNF-α	Activate NF-κB → Induce Claudin 1 expression	Induce gene expression and promote migration of GC cells	up
David [92]	TGF-β	Mediate Smad4 inactivation signal → EMT	Promote GC invasion and metastasis	up
Tauriello [93]	TGF-β	Promote immune escape	Promote GC invasion and metastasis	up
Ishimoto [9^94^]	TGF-β1	Enhance the motor capacity of fibroblasts	Promotes GC cells migration	up
Han [95]	IL-1	Up-regulate mirRNA-135b → Down-regulate mRNA of FOXN3 and RECK	Increase cell invasiveness and stem cell properties	up
Xuan [97]	IL-1α	Activate IL-1α/hypoxia signaling pathway	Promote tumor metastasis and cisplatin resistance	up
Zhang [9^99^]	IL-1β	Activate ERK pathway, Induce EMT	Promote GC invasion and metastasis	up
Yu [101]	IL-1β	Activate PI3K/S100A4 pathway	Promote tumor metastasis, increase stem cell activity	up
Wu [105]	IL-6	Activate JAK2/STAT3 pathway	Promote GC cells migration	up
Zhao [106]	IL-6	Activate JAK-STAT3-VEGF-C signaling pathway	Promote GC cells growth and invasion, promote lymphangiogenesis	up
Sánchez-Zauco [104]	IL-10	–	Diagnostic biomarker	up
Chen [1^110^]	IL-10	Activate c-Met/STAT3 pathway	Promote GC cells proliferation and migration	up
Zou [114]	IL-10	–	Predict GC occurrence	up
Wang [143]	IL-11	Activate STAT3/ERKsignaling pathway → Up-regulate MUC1	Promote GC invasion and metastasis	up
Li [115]	IL-12	–	Anti-tumor immune effect	down
Xing [117]	IL-12	Activate NKp30/MAPK → NK cells recruitment	Slow tumor development	down
Zhang [119]	IL-17	Gene polymorphisms have a synergistic effect on chronic Hp infection	Increased risk of GC	up
Meng [120]	IL-17	–	Promote tumor angiogenesis	up
Wu [121]	IL-17	Activate STAT3 mediated signaling pathway → Up-regulate VEGF	Promote intratumor vessels formation, increase intratumor microvessels density	up
Gunjigake[123]	IL-17A	–	Promote peritoneal metastasis and fibrosis formation	up
Kim [124]	IL-18	Activate JNK pathway → Increase TSP-1 expression	Promote intratumor angiogenesis	up
Tomita [127]	IL-18	Promote IFN-γ secretion → Increase Th1 response	Induce a persistent inflammatory response	up
Chen [128]	IL-18	Promote the secretion of TNF-α and IFN-γ → Enhance cytotoxicity and synergistic effect with IL-12	Anti-tumor effect	down
Ji [144]	IL-22	Activate IL-22R1/AKT/MMP-9 signaling pathway	Promote GC invasion and metastasis	up
Tsai [145]	IL-32	Activate related signaling pathway of AKT, β-cateninand HIF-1α	Promote GC invasion and metastasis	up
Yu [146]	IL-33	Activate ST2-ERK1/2 pathway → Up-regulate MMP-3 and IL-6	Promote GC invasion and metastasis	up
Kuai [130]	CXCL8	Activate NF-κB and Akt signaling pathway → Increase the expression of ICAM-1, VCAM-1 and CD44	Promote GC cells adhesion, migration and invasion, promote oxaliplatin resistance	up
Lin [134]	CXCL8	Induce PD-L1 + macrophages	Formation of immunosuppressive microenvironment in GC	up
Park [135]	CXCL5	–	–	up
Yasumoto [136]	CXCL12	Activate CXCL12/CXCR4 axis	Promote GC peritoneal metastasis	up
He [137]	CXCL13	–	–	up
Wang [139]	CCL5	–	Promote GC peritoneal metastasis	up
Tao [140]	CCL2	–	–	up
Hwang [141]	CCL7	–	Promote lymph node metastasis	up
Hwang [141]	CCL21	–	Promote lymph node metastasis	up
Wei [142]	CCL22	Recruit Treg cells → Suppress immune response	Promote GC peritoneal metastasis	up

### TGF-β

TGF-β is a multidirectional and pleiotropic cytokine, which is involved in regulating cell proliferation, differentiation, and apoptosis. It plays a dual role in tumor development. On the one hand, in the precancerous stage, it can inhibit cell proliferation and induce apoptosis through various signal pathways, so as to inhibit the occurrence of tumor. On the other hand, in advanced tumors, it can promote the invasion and metastasis of tumor cells by regulating the immune system and tumor microenvironment (95). TGF-β can promote EMT by mediating the Smad4 inactivation signal, so as to transform polar epithelial cells into active mesenchymal cells. This enables epithelial cells to acquire the capacity for invasion and migration (96). In addition, the high expression of TGF-β in the tumor microenvironment can promote tumor immune escape, strengthen the adhesion and invasion of cancer cells, and affect the prognosis of patients (97).

TGF-β1 is the most widely distributed subtype of TGF-β and one of the cytokines most closely related to tumors. Activation of TGF-β signal transduction can enhance the motility of fibroblasts and induce invasiveness of GC cells (98). Serum TGF-β1 level was reportedly significantly up-regulated in GC patients. These levels were significantly higher the levels in patients with EGC, and was positively correlated with disease severity (99) (**Table 2**).

### IL-1

IL-1 is a very important growth factor. It is often abnormally expressed in the pathological processes of inflammation, tumors, transplantation rejection, and immune diseases, and directly affects the occurrence, development, and prognosis of disease. IL-1 is located on human chromosome 2q13-21. Various types of tumor cells can produce IL-1. IL-1 is important in the growth of tumors. It can promote tumorigenesis by regulating the proliferation and differentiation of cells, and promotes the metastasis of cancer cells by regulating the expression of glycoproteins on the surface of tumor cells and conduction pathways. In addition, IL-1 promotes the occurrence of inflammation-related GC in mice by up-regulating mirRNA-135b (100). IL-1α and IL-1β are two forms of IL-1. IL-1α was reportedly up-regulated during hypoxia and was positively correlated with GC stage, lymph node metastasis, and cisplatin resistance (101). The IL-1α/hypoxia axis may be a valuable target for the diagnosis and treatment of GC.

IL-1β is a proinflammatory cytokine encoded by *IL-1B*. In GC patients, the expression of IL-1β is significantly up-regulated. IL-1β promotes the development of GC by participating in precancerous lesions and hypogastric acid secretion after *H. pylori* infection (102). Zhang et al. found that the expression of IL-1β was significantly increased in GC cells, which induced ERK pathway activation and EMT in GC cells (103). IL-1β can mediate the decrease of gastric acid secretion caused by *H. pylori* infection and can lead to the redistribution of H. *pylori* in the gastric body. The ensuing development of gastric atrophy further aggravates the colonization of *H. pylori* and other bacterial infections, resulting in the continuous accumulation of toxins and inflammatory products produced by bacteria (104). Therefore, Il-1 β may play a leading role in the pathogenesis model of “chronic gastritis-gastric mucosal atrophy-atypical hyperplasia gastric cancer”. Yu et al. confirmed that IL-1β could promote the nuclear translocation of S100A4 in MGC803 GC cells through the PI3K pathway, so as to enhance the stem cell properties of these cells (105). In addition, the occurrence of GC is a multi-gene, multi-factor, multi-stage and multi-path process resulting from the combined action of genetics and epigenetics. Epigenetic changes are heritable changes that do not depend on DNA sequence changes. These changes have a major role in the initiation and progression of tumors. Hmadcha et al. found that IL-1β activated a new gene silencing pathway through nitric oxide that was modified by DNA methylase and CpG island methylation (106). Qian et al. found that IL-1β could induce methylation of E-cadherin gene promoter in gastric mucosal cells (107) (**Table 2**).

### IL-6

IL-6 is encoded by a gene located on chromosome 7. The region contains 5 exons and 4 introns. IL-6 is a pleiotropic cytokine that is important in inflammation, bone metabolism, and the occurrence and development of tumors. Sánchez-Zauco et al. significantly correlated the level of serum IL-6 with the occurrence of GC (108). Wu et al. described that IL-6 can enhance the migration of GC cells by activating the Janus kinase 2/signal transducer and activator of transcription 3 (JAK2/STAT3) pathway (109). Zhao et al. confirmed that IL-6 could increase the expression levels of JAK, STAT3, phosphorylated-STAT3 and VEGF-C in GC cells, and can promote the growth, invasion, and lymphangiogenesis of GC through the JAK/STAT3/VEGF-C signaling pathway (110). Furthermore, IL-6 can stimulate GC cells to produce a large amount of VEGF, which promotes the formation of new blood vessels and affects tumor invasion and progression. In addition, IL-6 can promote the invasiveness of GC cells, which may be related to omental metastasis of GC. Wu et al. further found that CAF activates the JAK2/STAT3 pathway of GC cells by secreting IL-6, which promotes the migration and EMT of GC cells. Silencing the expression of IL-6 in CAF can inhibit the peritoneal metastasis of tumors induced by CAF *in vivo* (109). IL-6 is highly expressed in the serum and cancer tissues of GC patients and is related to the depth of cancer invasion and degree of differentiation. These findings indicate the potential of IL-6 as a marker for GC and an indicator of poor prognosis (**Table 2**).

### IL-10

IL-10 is an inflammatory cytokine located on chromosome 1q31-32. It has the dual abilities of regulating immune suppression and immune stimulation. It mainly has an immunosuppressive role in the inflammatory response (111). IL-10 inhibits Th1 cells, Th2 cells, and B lymphocytes. This actions inhibit the production of proinflammatory cytokines and down-regulate the inflammatory response (112). In addition, IL-10 is considered to be a protective factor in *H. pylori* associated gastritis (113). As well, in the occurrence and development of cancer, IL-10 promotes the growth and proliferation of cancer cells. Sánchez-Zauco et al. found that the level of IL-10 in EGC patients was significantly increased, indicating that it may be a diagnostic biomarker of GC (108). Chen et al. found that IL-10 promoted the occurrence and development of GC by activating the C-MET/STAT3 signaling pathway (114). Most studies to date have been concerned with the promoter region of IL-10. This region mainly includes three polymorphic loci: -108 (A-G), -819 (T-C), and -592 (A-C). Zhuang et al. demonstrated the close relationship between gene polymorphism of IL-10 and the incidence rate of GC in Asian carriers (115). Sugimoto et al. studied the relationship between Japanese GC patients and the three polymorphic loci mentioned above. IL-10-819 and IL-10-592 increased the risk of GC (116). However, in Latin American GC patients, only the IL-10-592C/A single nucleotide polymorphism was associated with the incidence of GC (117). In addition, the expression level of IL-10 in AGC patients was significantly increased. Zou et al. also pointed out that the increase of IL-10 may be a risk factor for tumor enlargement, and may help to predict the occurrence and development of GC (118). However, the increased expression of IL-10 in EGC may be due to the secretion by cancer cells themselves or the product of anti-tumor immune effect. At present, there is a lack of research on IL-10 in GC patients. The mechanism of IL-10 in promoting the development of GC in patients is not clear (**Table 2**).

### IL-12

IL-12 is secreted by dendritic cells, macrophages, neutrophils, and B lymphocytes. It can stimulate the growth and differentiation of T cells, which identify allogeneic cells in the immune response of the body. The level of IL-12 was reportedly significantly increased in EGC patients and had an anti-tumor effect (119). However, another study documented a significant decrease in IL-12 during the progression of GC. The potential mechanism involved the significant increase in the level of GOLPH2 and the ensuing down-regulated expression of IL-12A and inhibited expression of TNF-α and interferon-gamma (IFN-γ). Simultaneously, the effect of Th1 was inhibited and the anti-tumor effect lessened (120). In a mouse GC cell line model, IL-12 sensitized the cells to natural killer (NK) cell lysis by activating the NKp30/MAPK/IL-12 pathway. Systemic administration of IL-12 combined with intratumoral injection of anti-HLA-I in tumor-bearing mice reportedly increased the recruitment of NK cells in transplanted tumors, made them sensitive to NK killing, and slowed down the progression of GC (121). Therefore, early identification of IL-12 changes may provide a target for GC prevention and treatment (**Table 2**).

### IL-17

IL-17 is a proinflammatory cytokine mainly produced by Th17 cells. IL-17 induces the secretion of early immune mediators that stimulate the accumulation of inflammatory response cells at the injury site. IL-17 is significantly expressed in a variety of solid tumors. The level of IL-17 in GC patients is significantly higher than that in healthy individuals (122). IL-17A rs2275913 G > A and IL-17F rs763780 T > C polymorphisms increase the risk of GC and act synergistically with *H. pylori* infection (123). Meng et al. reported the participation of IL-17 in the occurrence and development of GC by promoting the angiogenesis of the tumor microenvironment (124). Wu et al. confirmed that IL-17 was usually overexpressed in patients with GC and up-regulated the expression of VEGF to promote angiogenesis and increase the microvessel density in the tumor, which was conducive to early metastasis of the tumor (125). IL-17 can also promote the occurrence and development of tumors by inhibiting apoptosis and regulating the immune response. In addition, IL-17 participates in the occurrence and development of GC, but also predicts the prognosis of GC patients. The high expression of IL-17 in GC patients has been associated with lower 5-year survival rate (126). IL-17A mainly comes from Th17 cells and is related to tumor occurrence, proliferation, and angiogenesis. IL-17A can promote the peritoneal dissemination of GC and the formation of fibrosis (127). Therefore, IL-17, as a specific tumor marker, can provide a good basis for clinical early diagnosis of occurrence and development of GC, and is very important for early treatment of GC (**Table 2**).

### IL-18

IL-18, encoded by a gene located in 11q2 2-q2, is derived mainly from the monocyte macrophage system, small intestinal epithelial cells, and spleen. IL-18 plays a role by binding to its receptor and is an important immune regulatory factor. The many biological functions of IL-8 include promoting the production of IFN-γ, IL-2 and granulocyte macrophage-colony stimulating factor by T cells and NK cells, enhancing the expression of Fas ligand in Th1 cells and NK cells, promoting the development of Th1 cells, and activating activator protein 1, nuclear factor of activated T cells, and signal transduction and transcription activating factors. IL-18 plays a dual role in the occurrence and development of GC. On the one hand, IL-18 can promote the occurrence and development of GC. IL-18 can increase the expression of thrombospondin 1 by activating the JNK pathway, which increases tumor angiogenesis (128). IL-18 can also promote the invasion and metastasis of GC cells by inhibiting the production of anti-tumor factors (129). Clinical evidence indicates an association between lower IL-18 levels in patients with GC and higher rates of postoperative survival (130). On the other hand, IL-18 has anti-tumor effects. A study described significantly higher levels of IL-18 mRNA in patients with *H. pylori* infection than in noninfected patients. IL-18 promoted secretion of IFN-γ by gastric mucosal cells, thereby promoting the Th1 response (131). Another study confirmed that IL-18 promoted the secretion of TNF-α and IFN-γ, which synergistically enhanced the cytotoxic function of tumor-infiltrating lymphocytes with IL-12 in GC, and produced an anti-tumor effect (132) (**Table 2**).

### Chemokines

Chemokines are proteins secreted by cells under pathological conditions. Endothelial cells, fibroblasts, epithelial cells, leukocytes and tumor cells can secrete chemokines. These include C-C motif, X-C motif, C-X-C motif, and C-X3-C motif chemokines. The CXCL chemokine family is important in the pathogenesis of GC and can be used as a marker of the occurrence and development of GC. CXCL8, also known as IL-8, is mainly produced by monocyte macrophages, lymphoid cells, and endothelial cells. It is an endogenous and multi-source cytokine that functions in chemotaxis and activation of leukocytes, and is involved in the occurrence and development of tumors. CXCL8 is highly expressed in GC patients (133). CXCL8 can increase the activities of NF-κB and AKT, increase expression of adhesion molecules ICAM-1, VCAM-1, and CD44, promote adhesion, migration, and invasion of GC cells, and is involved in the development of resistance to oxaliplatin (134). In addition, CXCL8 is closely related to the adhesion and migration of GC cells. When these cells are exposed to *H. pylori*, they produce CXCL8, which in turn promotes the progression of GC through autocrine and paracrine mechanisms (135, 136). Therefore, inhibiting the expression of CXCL8 may reduce the adhesion, migration, and invasion of GC cells. Wu et al. confirmed that the level of CXCL8 was significantly correlated with the depth of venous invasion and lymphatic invasion, which may be an independent prognostic factor of GC (137). Li et al. confirmed the participation of CXCL8 in the formation of the immunosuppressive microenvironment of GC by inducing PD-L1 positive macrophages (138). Therefore, CXCL8 inhibitors may drive an anti-tumor response and could be a novel approach in treating GC patients. CXCL5 is mainly expressed on the surface of lymphocytes, macrophages, and renal tubular epithelial cells. It is also expressed in GC. The gene for CXCL5 is located in 17q12. Park et al. reported the higher concentration of CXCL5 in AGC patients than in patients with benign tumors (139). The overexpression of CXCL5 was positively correlated with the stage of GC, especially N stage. These results suggest a role for CXCL5 in the progression of GC, especially in lymph node metastasis. CXCL12, also known as matrix derived factor 1, exhibits strong chemotaxis to lymphocytes and is found in GC with lymph node metastasis. CXCR4 is a specific receptor of CXCL12. Yasumoto et al. found that the expression of CXCR4 was significantly correlated with the occurrence of peritoneal metastasis of GC, with the CXCL12/CXCR4 axis having an important role in peritoneal metastasis of GC (140). In addition, high expression of CXCR4 predicts a poor prognosis in GC patients. He et al. confirmed that the up-regulation of CXCR4 expression in tumors was associated with poor OS in patients with GC (141). In addition, CXCL13 is also considered to be an effective index to judge the prognosis of GC patients (142).

The CCL chemokine family also plays an important role in the pathogenesis of GC. Wang et al. showed that CCL5 is a good biomarker of occult GC peritoneal metastasis and has diagnostic value for GC peritoneal metastasis (143). Tao et al. described the elevated expression of CCL2 in GC specimens. The OS rate of GC patients with elevated CCL2 expression was lower than that of individuals with low CCL2 expression. The study findings suggest that CCL2 can be used as an independent prognostic marker of GC (144). Hwang et al. found that CCL7 and CCL21 were overexpressed in GC and were related to lymph node metastasis and poor prognosis (145). Wei et al. found that the level of CCL22 in patients with peritoneal metastasis of GC was significantly higher than that in patients without metastasis (146). Highly expressed CCL22 can recruit T regulatory cells in the tumor microenvironment, inhibit an immune response, and participate in mediating peritoneal metastasis of GC.

The collective evidence indicates that chemokines are important in the occurrence and development of GC. Comprehensive studies of chemokines and their receptors will further the early diagnosis and prognosis of GC (**Table 2**).

### Others

IL-11 is a member of glycoprotein-130 (gp-130) cytokines. Wang et al. reported the up-regulated expression of mucin 1 protein cancer-associated fibroblasts *via* the IL-11-STAT3/ERK signaling pathway, and the important role of IL-11 in the progression of the GC microenvironment (147). Targeted treatment of IL-11 can be indirectly effective in the treatment of GC by affecting interstitial fibroblasts. IL-22 is an important inflammatory cytokine in the IL-10 family, with a role through the IL-22 receptor. The expression of IL-22 was reportedly increased significantly in GC patients, suggesting that it may be related to the occurrence of GC. The authors also described that IL-22 could regulate the IL-22R1/AKT/MMP-9 signaling pathway and enhanced the migration and invasion of GC cells by binding to its receptor (148). IL-32 is a proinflammatory cytokine characterized by induction of NF-κB activation. Tsai et al. confirmed that the expression of IL-32 was enhanced in patients with GC and positively correlated IL-32 with the severity of GC. Furthermore, IL-32 can promote tumor migration and invasion by increasing the activation of AKT, β-catenin, and hypoxia-inducible factor 1-alpha related signaling pathways (149). IL-33 is a member of the IL-1 superfamily and is a multifunctional cytokine released during inflammatory response, biomechanical stress, or necrotic cell death. The significant increase of IL-33 in GC patients has been described, with a close relationship to the depth of invasion and distant metastasis of GC. Yu et al. found that IL-33 could stimulate the secretion of MMP-3 and IL-6 through the ST2-ERK1/2 pathway, thereby promoting the invasion and migration of GC cells (150). IL-35 is an anti-inflammatory cytokine produced by T regulatory cells, with a unique immunomodulatory function. IL-35 is highly expressed in GC and is a potential biomarker for clinical diagnosis of GC (151) (**Table 2**).

## Outlook

Guiding individual therapy of GC is an important goal and active area of research. Liquid biopsy is minimally invasive, economical, and time-saving. As a valuable alternative to tissue biopsy, liquid biopsy has gradually attracted the attention of researchers. Due to its use for relatively personalized diagnosis and treatment, liquid biopsy is important in precision medicine. However, the low concentration of relevant biomarkers in the blood hinders the detection sensitivity of this method. Also, the separation technology is not yet widely applicable, with consensus not yet reached for some standards. More rigorous studies must explore the promising potential of liquid biopsy technology. These data will inform the increasingly accurate and rapid diagnosis and treatment of various tumors, including GC.

## Author contributions

XM designed the article form and wrote the manuscript. XL and KO consulted and browsed the literature. LY revised the manuscript. All authors read and approved the final manuscript.

## Funding

This work was funded by the Beijing CSCO Clinical Oncology Research Foundation (Y-HH202102-0308).

## Conflict of interest

The authors declare that the research was conducted in the absence of any commercial or financial relationships that could be construed as a potential conflict of interest.

## Publisher’s note

All claims expressed in this article are solely those of the authors and do not necessarily represent those of their affiliated organizations, or those of the publisher, the editors and the reviewers. Any product that may be evaluated in this article, or claim that may be made by its manufacturer, is not guaranteed or endorsed by the publisher.
